# The Effect of Body Mass Index on Outcome of Abdominoplasty Operations

**Published:** 2016-09

**Authors:** Wagih Ghnnam, Ashraf Elrahawy, Magdy EL Moghazy

**Affiliations:** 1 Department of General Surgery, Mansoura Facculty of Medicine, Mansoura University, Egypt;; 2 Department of Plastic Surgery, Menoufia Faculty of Medicine, Menoufia University, Egypt

**Keywords:** Abdominoplasty, Body Mass Index, Complications, Seroma, Patient satisfaction

## Abstract

**BACKGROUND:**

Increased body mass index (BMI) increase the incidence of seroma formation and wound infection rates and subsequently increases wound dehiscence and ugly scar formation following abdomenoplasty and body contour surgery and also many other aesthetic and plastic surgery. The aim of this study was to determine the effect of BMI on the outcome of abdominoplasty operation.

**METHODS:**

We carried out a prospective study of all patients who underwent abdominoplasty at our institution. Patient were divided into two groups. Group I were subjects with body mass index <30 kg/m^2^ while group II were patients with body mass index >30 kg/m^2^. Demographics and complications (minor and major) were recorded.

**RESULTS:**

Sixty seven patients were enrolled. Group I were 32 patients with a mean age of 35.71 and group II 35 patients with mean age of 36.26 years. Seroma formation, wound complications, prolonged hospital stay and complications were significantly more in group II.

**CONCLUSION:**

We found that increased BMI significantly increased operative time, hospital stay, drainage duration and drainage amount. Our findings showed that obesity alone could increase the incidence of complications and poor outcome of abdominoplasty.

## INTRODUCTION

Abdominoplasty is an extensive surgical operation, usually followed by a significant number of local and general complications. Some studies indicate that the risk of severe complications, including mortality, ranges from 1 in 617 to 1 in 2,320 cases.^1^ Hematoma and seroma formation in surgical wounds had negative effects on wound healing and subsequent morbidity to patients. The etiology is multifactorial, involving inadequate hemostasis, lymphatic disruption, shearing between tissue surfaces, creation of surgical dead space, and systemic coagulopathy.^2^ Seroma formation is a common post abdominoplasty complication, resulting in significant patient morbidity. The incidence is particularly high where it is reported to occur in 10% to 57% of the patients.^1^^-^^2^ The higher incidence rates are reported in obese patients. 

Body mass index (BMI) is the traditional way of assessing patients for body contouring surgery and has been shown to have a predictive value for surgical complications.^3^ BMI has been identified in many studies as a negative predictor of wound healing in abdominal surgery and in breast reconstruction surgery.^4^ Abdominoplasty has a higher complication rate than other aesthetic procedures. Despite a contemporary history of about 50 years, the basic steps of extensive undermining, skin resections, muscle plication and umbilical transposition have remained unchanged. Although the complication rates have dropped, case series with high complication rates are still reported, striking a note of caution. These complications lead to dissatisfaction, prolonged convalescence, unforeseen expenses, physical and psychological suffering and at rarely times, they may be dangerous or fatal.^5^^-^^8^ The aim of our work was to study the effect of BMI on the outcome and complications of abdominoplasty.

## MATERIALS AND METHODS

**Fig. 1a, b, c:**
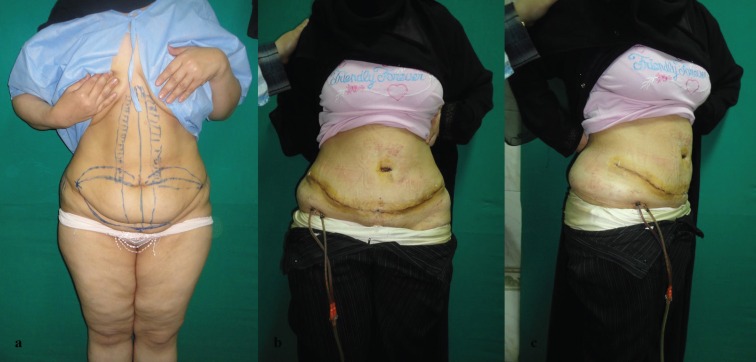
Preoperative and postoperative picture of abdominoplasty case with BMI <30 kg/m^2^.

**Fig. 2a, b, c:**
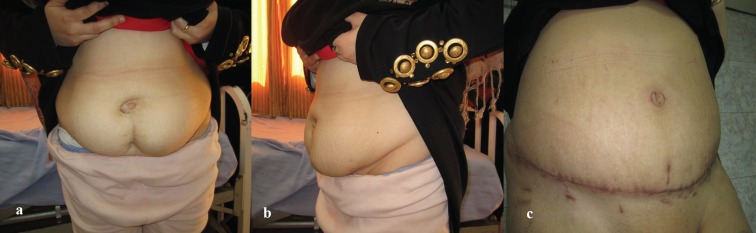
Preoperative and postoperative picture of abdominoplasty case with BMI <30 kg/m^2^.

**Fig. 3a, b:**
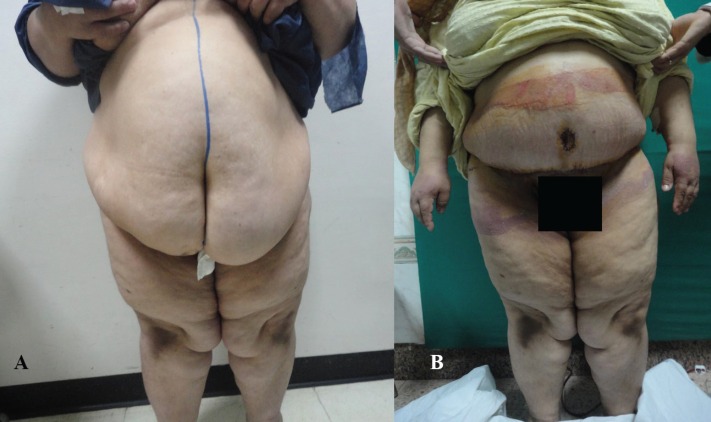
Preoperative and postoperative picture of abdominoplasty case with BMI >30 kg/m^2^.

**Fig. 4a, b:**
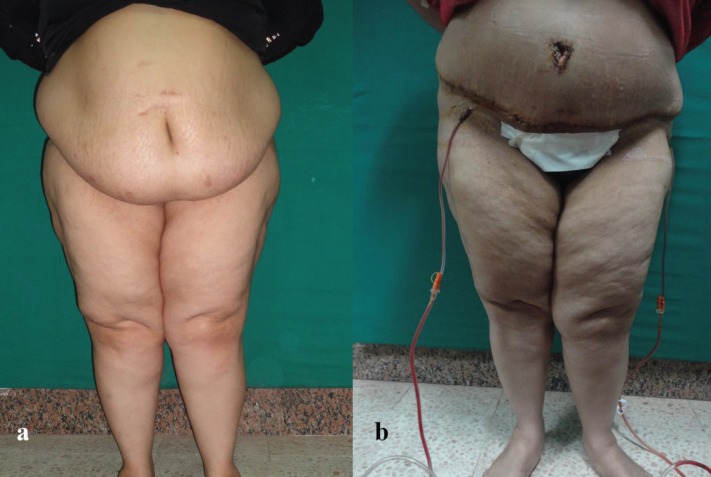
Preoperative and postoperative picture of abdominoplasty case with BMI >30 kg/m^2^.

**Fig. 5a, b:**
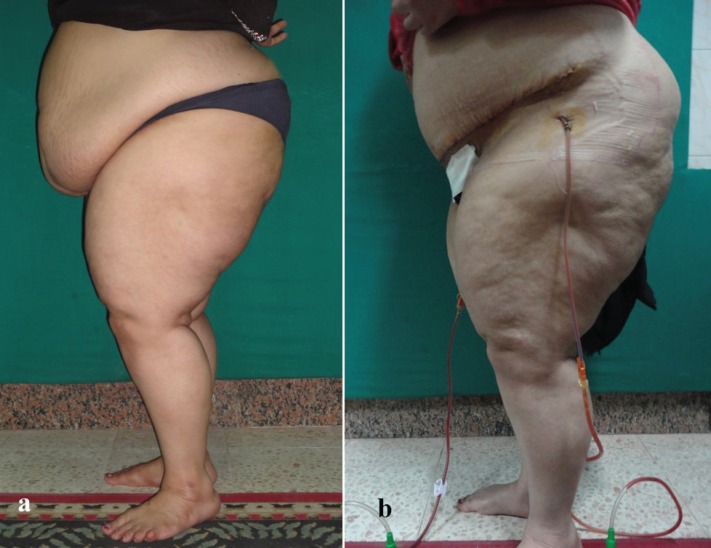
Preoperative and postoperative picture of abdominoplasty case with BMI >30 kg/m^2^.

**Fig. 6a, b:**
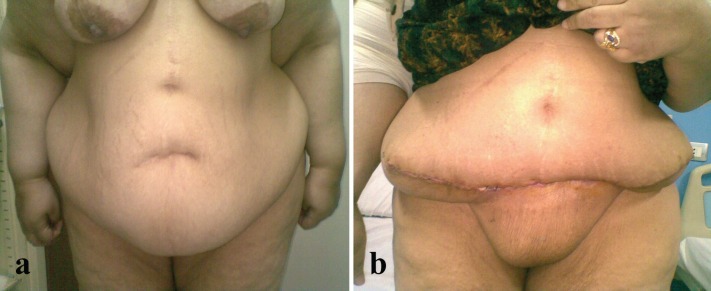
Preoperative and postoperative picture of abdominoplasty case with BMI >30 kg/m^2^

Between January 2011 and January 2015, sixty seven patients were enrolled while all were females aged 26–47 years. All patients had mainly infra umbilical fat deposits, skin excess in the infra umbilical segments and some of them had ventral hernia and diastasis of rectus muscles. Patients were divided into two groups. Group I consisted of 32 patients with BMI less than 30 kg/m^2^ and Group II were 35 patients with BMI more than 30 kg/m^2^. All patients were subjected to traditional abdominoplasty as sescribed before^9^ (Figures 1-6).

**Fig. 7:**
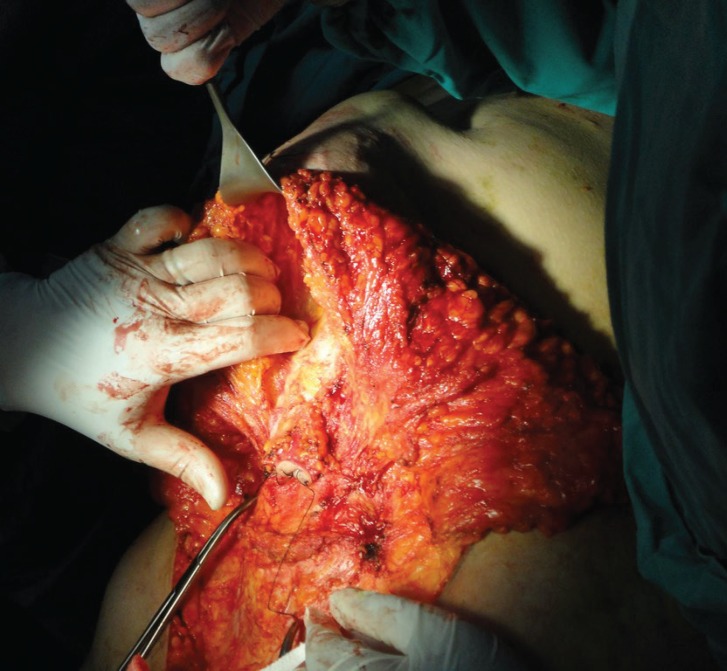
Dissection of the umbilicus from the flap

Preoperative markings were crucial to successful surgery and to get desired symmetrical results. Patients were marked preoperatively in the standing position, and a transverse line was made just above the pubic hair extending laterally 7 to 18 cm in each direction towards the anterior superior iliac spine. Skin incision following the previously determined markings at the lower abdomen was done, sometimes a vertical incision from the umbilicus to the pubis was added to facilitate dissection. The umbilical scar was isolated (Figure 7) and dissection of the deep tissues was performed with electro cautery. Below the umbilicus, the plane of dissection was just beneath the level of Scarpa’s fascia leaving a thin fatty layer with its connective tissue and lymphatic vessels especially laterally.

The umbilicus was transposed and excess skin was removed. The flap was pulled down to estimate the amount of skin to be resected, and then the redundant skin and fat were excised. In some cases, aponeurotic plication was performed from the epigastrium to the pubis using interrupted 0 prolene suture reinforced with continuous running 0 Prolene. In cases with abdominal wall hernia, on lay prolene mesh was fixed to the abdominal wall with 0 prolene sutures and two suction drains were exteriorized. The wound was closed (tension free) in two layers, vicryl 3-0 stitches were applied to fix the scarpa`s fascia of the superior abdominal flap to the inferior scarpa fascia border. 

The 3–0 prolene subcuticular stitches were performed in most of cases and few cases were closed with skin stapler. Patients were allowed to ambulate immediately at the night of surgery. Suction drains were removed before patients were discharged when the output is less than 50 ml of serous fluid per day. Statistical analysis was performed using SPSS statistical software for windows (the 20.0 version, Chicago, IL, USA). Unpaired student t test was used for comparison of two groups for age, BMI, pannus weight and hospital stay. Chi square and Fisher exact tests were used to compare the 2 groups in incidence of complications and patient satisfaction. P values less than 0.05 were considered significant.

## RESULTS

There was no significant differences between group I and group II regarding age of the patients (Table 1). The median time spent in the operating room was 90 minutes for group I and 155 minutes for Group II (*p*=0.01). The weight of tissue resection varied from 790 g to 4.68 kg; that were significantly more in group II patients (median 3310 g) when compared to Group I (median 1835 g) (*p*=0.01). The patients were hospitalized for 3-21 days with a median of seven days in both groups. There was a significant difference in drainage time and output for both groups with a median of six days for all patients and a median output of 520 ml and 845 ml in group I and group II, respectively (*p*=0.01) (Table 1). The overall complication rate was 31.3%. Eleven patients (16.4%) showed major complications that required surgical intervention or aspiration and 14.9% revealed minor complications that did not need any intervention. No patients died or developed serious complications such as deep venous thrombosis or pulmonary embolism. 51.4% of group II patients had a complication, whereas only 9.4% of group I demonstrated a complication (*p*=0.01, Table 2).

**Table 1:** General characteristics and results of both groups, n=67 (NS: Not significant

	**Group A (n=32)**	**Group B (n=35)**	**Total (n=67)**	***p*** ** value**
Mean age, years (Range)	35.71±5.31 (25-47)	36.26±4.57 (27-44)	36±4.9 (25-47)	0.65
Body mass index, Kg/m^2 ^Mean (Range)	28.4±1.39 (25.3-30)	35.56±2.89 (31-41.5)	32.14±4.23 (25.3-41.5)	0.0001
Previous abdominal surgeries (%)	7 (21.9)	9 (25.7)	16 (23.9)	0.9382
Mean operative time minutes (range)	93.9±12.8 (80-120)	152.1±22.3 (100-180)	124.3±34.5 (80-180)	0.0001
Pannus weight, g Mean (Range)	1699.25±506.79 (790-2530)	3355±738 (1870-4680)	2562.36±1048.44 (790-4680)	0.0001
Drain duration, day Mean (Range)	4.96±1.73 (3-10)	7.97±3.4 (3-18)	6.54±3.11 (3-18)	0.0001
Drain output, ml Mean (Range)	587.34±246 (210-1250)	1133.57±702 (350-2670)	872.69±599 (210-2670)	0.0001
Hospital stay, day Mean (Range)	5.94±1.92 (3-12)	10±3.69 (5-21)	8.05±3.6 (3-21)	0.0001

**Table 2:** Complications in the study groups

**Complications **	**Group A (n=32)**	**Group B (n=35)**	**Total (n=67)**	***p*** ** value**
Seroma number (%)	1 (3.13)	4 (11.43)	5 (7.46)	0.4096
Wound dehiscence number (%)	0	3 (8.57)	3 (4.48)	0.2700
Wound infection number (%)	1 (3.13)	2 (5.71)	3 (4.48)	0.9336
Ugly scar number (%)	0	2 (5.71)	2 (2.99)	0.5146
Dog ears number (%)	0	2 (5.71)	2 (2.99)	0.5146
Loss of sensation number (%)	1 (3.13)	4 (11.43)	5 (7.46)	0.2422
Dissatisfaction number (%)	1 (3.13)	5 (14.29)	6 (8.96)	0.2123

The most frequent complication was patient’s dissatisfaction in 6 patients followed by loss of sensation and seroma formation in five patents. Seroma occurred in five patients of whom three required aspiration. All five patients had drains that were removed on the third to seventh day (Table 2). Considering local complications, Group I showed a decrease in the incidence of seroma, partial wound dehiscence, skin edge necrosis and loss of sensation in lower abdominal skin in comparison to group II (Table 2). One patient in group I developed seroma compared to 4 patients in group II. Seroma developed 1 to 3 weeks postoperatively and were solved with repeated syringe aspiration in 4 cases and one patient in group I resolved spontaneously with antibiotic and anti-inflammatory drugs. One patient in group I developed minor wound infection that responded to antibiotics. Partial wound dehiscence did not occur in group I patients, compared to three patients in group II. Dehiscences were treated conservatively by repeated dressing in two patients and debridement and later skin graft in the third patient (Table 2).

## DISCUSSION

During abdominoplasty, all disorders of the abdomen must be treated to reinstate the harmony of the body contour. Abdominoplasty operations vary in scope, and are frequently subdivided into categories. Depending on the extent of the surgery, a complete abdominoplasty can take 1 to 5 hours. In our study, it took 2 to 3 hours in conjunction with hernia repair in some cases.^10^ We found that increased BMI significantly increased operative time, hospital stay, drainage duration and drainage amount (Table 1), and also it increased minor and major local complications although statistically insignificant (Table 2). It is difficult to compare current literature due to the different definitions used for minor and major complications. 

It has been reported that patients with a BMI<25 kg/m^2^ had a 3.3% risk of developing minor complications (infection, seroma, minor wound problems) and a 6.7% risk of developing a major complication (significant wound healing problems, dehiscence, readmission, re-operation, tissue necrosis, death).^11^ At a BMI of 25-30 kg/m^2^, a total of 18.2% developed minor and 13.6% developed major complications. For patients with a BMI>40 kg/m^2^, the numbers were 46.9% and 43.8%, respectively. In another study, it was found that BMI may impact complications in single-procedure cases.^12^

The incidence of complications following abdominoplasty may be as high as 80% in obese patients. Because of the extent of undermining and the thick abdominal pannus encountered in the majority of these patients, they are more susceptible to seromas, hematomas, and wound dehiscence .The use of drains is a necessity any time there is undermining. A suction drain on each side with puncture sites concealed at length from incisions should remain with constant suction until the daily output of each drain drops below 20 mL.^13^

In spite of the progress in the abdominoplasty techniques, a significant complication rate is still associated with abdominoplasty including flap necrosis, seroma, hematoma, infections, wound dehiscence, and delayed healing of wound. Avoidance of these complications begins with proper choice of the patient and matching the procedure to the type of abdominoplasty.^14^ The abdomen is examined to identify the amount of excess fat to be removed, status of the muscles and extent of midline divarication, presence of umbilical or other ventral abdominal hernias, extent of upper abdominal skin laxity, native position and orientation of the umbilicus, subcostal angle and the vertical distance between costal margin and the upper border of iliac bone.^15^

The incidence of complications following abdominoplasty may be as high as 80% in obese patients. Because of the extent of undermining and the thick abdominal pannus encountered in the majority of these patients, they are more susceptible to seromas, hematomas, and wound dehiscence .The use of drains is a necessity any time there is undermining. A suction drain on each side with puncture sites concealed at length from incisions should remain with constant suction until the daily output of each drain drops below 20 mL.^16^

In an extensive survey of 10,940 abdominoplasties performed by 958 plastic surgeons from all over the world, the complication was thromboembolism (1.9%).^17^ In our study, we did not have thromboembolism, because all patients were female favoring the conclusion that, women are less hypercoagulative than men in the postoperative period, suggesting that women have limited protection from the development of thromboembolic complications. In the literature, reports of wound infection, dehiscence and general poor scarring range from 0.9% to 8 % .^18^^-^^21^ We had partial wound dehiscence and skin necrosis in 3 patients (4.48%) and bad scarring in two patients (2.99%).

The general satisfaction rate after abdominoplasty is still very high,^22^ and aesthetic dissatisfaction is commonly voiced by patients. Based on our knowledge, there were no published studies on this complication and we showed a comparable satisfaction rate of 91.1% in all groups while dissatisfaction was more common in Group II patients (Table 2). We found that increased BMI significantly increased operative time, hospital stay, drainage duration and drainage amount. So our findings showed that obesity alone could increase the incidence of complications and poor outcome of abdominoplasty.

## CONFLICT OF INTEREST

The authors declare no conflict of interest.

## References

[1] Momeni A, Heier M, Bannasch H, Stark GB (2009). Complications in abdominoplasty: a risk factor analysis. J Plast Reconstr Aesthet Surg.

[2] Chaouat M, Levan P, Lalanne B, Buisson T, Nicolau P, Mimoun M (2000). Abdominal dermolipectomies: Early postoperative complications and long-term unfavorable results. Plast Reconstr Surg.

[3] Rangaswamy M (2013). Minimizing complications in abdominoplasty: An approach based on the root cause analysis and focused preventive steps. Ind J Plast Surg.

[4] Van Uchelen J H, Werker P M N, Kon M (2001). Complications of abdominoplasty in 86 patients. Plast Reconstr Surg.

[5] Alderman AK, Collins ED, Streu R, Grotting JC, Sulkin AL, Neligan P, Haeck PC, Gutowski KA (2009). Benchmarking outcomes in plastic surgery: National complication rates for abdominoplasty and breast augmentation. Plast Reconstr Surg.

[6] Brauman D, Capocci J (2009). Liposuction abdominoplasty: An advanced body contouring technique. Plast Reconstr Surg.

[7] Beer GM, Wallner H (2010). Prevention of seroma after abdominoplasty. Aesthet Surg J.

[8] Zuelzer HB, Ratliff CR, Drake DB (2010). Complications of abdominal contouring surgery in obese patients: Current status. Ann Plast Surg.

[10] Avelar J (2006). Abdominoplasty combined with lipoplasty without panniculus undermining: Abdominolipoplasty a safe technique. Clin Plast Surg.

[11] Au K, Hazard SW 3rd, Dyer AM, Boustred AM, Mackay DR, Miraliakbari R (2008). Correlation of complications of body contouring surgery with increasing body mass index. Aesthet Surg J.

[12] Coon D, Gusenoff JA, Kannan N, El Khoudary SR, Naghshineh N, Rubin JP (2009). Body mass and surgical complications in the post-bariatric reconstructive patient: analysis of 511 cases. Ann Surg.

[13] Rogliani M, Silvi E, Labardi L, Maggiulli F, Cervelli V (2006). Obese and non-obese patients: complications of abdominoplasty. Ann Plast Surgy.

[14] Grieco M, Grignaffini E, Simonacci F, Raposio E (2015). Analysis of Complications in Post-bariatric Abdominoplasty: Our Experience. Plast Surg Int.

[15] Jacobs JMS, Schechner S, Jacobs JS (2006). Abdominoplasty Following Massive Weight Loss. Semin Plast Surg.

[16] Mejia JA, Cárdenas Castellanos YA (2012). Extended abdominoplasty: Applications and a new classification system for abdominoplasty. Aesthet Plast Surg.

[17] Grazer EM, Goldwyn RM (1977). Abdominoplasty assessed by survey, with emphasis on complications. Plast Recontr Surg.

[18] Kim J, Stevenson TR (2006). Abdominoplasty, liposuction of the flanks, and obesity: analyzing risk factors for seroma formation. Plast Reconstr Surg.

[19] Spiegelman JI, Levine RH (2006). Abdominoplasty: a comparison of outpatient and inpatient procedures shows that it is a safe and effective procedure for outpatients in an office-based surgery clinic. Plast Reconstr Surg.

[20] Stewart KJ, Stewart DA, Coghlan B, Harrison DH Jones BM, Waterhouse N (2006). Complications of 278 consecutive abdominoplasties. Plast Reconstr Aesthetic Surg.

[21] Neaman KC1, Hansen JE (2007). Analysis of complications from abdominoplasty: a review of 206 cases at a university hospital. Ann Plast Surg.

[22] Staalesen T, Elander A, Strandell A, Bergh C (2012). A systematic review of outcomes of abdominoplasty. J Plast Surg Hand Surg.

